# Supplementary material for: Multi‐dataset identification of innovative feature genes and molecular mechanisms in keratoconus
Source: J Cell Mol Med. 2024 Sep 19;28(18):e70079. doi: 10.1111/jcmm.70079 (PMC11412914; doi:10.1111/jcmm.70079)
Supplement: Supplementary file 2 — Material S2. [file JCMM-28-e70079-s002.zip › 7.GSEA analysis/5.RNF217-AS1/KEGG_P53_SIGNALING_PATHWAY.html]

Details for gene set KEGG\_P53\_SIGNALING\_PATHWAY[GSEA]

|  || Dataset | RNF217-AS1.group.cls#h\_versus\_l |
| Phenotype | group.cls#h\_versus\_l |
| Upregulated in class | l |
| GeneSet | KEGG\_P53\_SIGNALING\_PATHWAY |
| Enrichment Score (ES) | -0.34732646 |
| Normalized Enrichment Score (NES) | -1.3098838 |
| Nominal p-value | 0.1880998 |
| FDR q-value | 0.39961386 |
| FWER p-Value | 0.946 |
Table: GSEA Results Summary

  

Fig 1: Enrichment plot: KEGG\_P53\_SIGNALING\_PATHWAY      
 Profile of the Running ES Score & Positions of GeneSet Members on the Rank Ordered List

  

| SYMBOL | TITLE | RANK IN GENE LIST | RANK METRIC SCORE | RUNNING ES | CORE ENRICHMENT || 1 | TP53AIP1 | na | 3290 | 0.279 | -0.0557 | No |
| 2 | THBS1 | na | 4580 | 0.231 | -0.0604 | No |
| 3 | TSC2 | na | 4977 | 0.218 | -0.0412 | No |
| 4 | MDM4 | na | 6753 | 0.160 | -0.0699 | No |
| 5 | CCNE1 | na | 7161 | 0.148 | -0.0608 | No |
| 6 | TP73 | na | 8239 | 0.122 | -0.0747 | No |
| 7 | BID | na | 8429 | 0.118 | -0.0636 | No |
| 8 | SIAH1 | na | 8692 | 0.112 | -0.0554 | No |
| 9 | CASP3 | na | 9912 | 0.088 | -0.0782 | No |
| 10 | SERPINB5 | na | 12796 | 0.045 | -0.1551 | No |
| 11 | GADD45A | na | 13174 | 0.040 | -0.1604 | No |
| 12 | IGF1 | na | 13325 | 0.038 | -0.1593 | No |
| 13 | CCNE2 | na | 14005 | 0.031 | -0.1746 | No |
| 14 | CCNG1 | na | 14257 | 0.028 | -0.1779 | No |
| 15 | MDM2 | na | 14406 | 0.026 | -0.1786 | No |
| 16 | APAF1 | na | 14427 | 0.026 | -0.1756 | No |
| 17 | PIDD1 | na | 14684 | 0.023 | -0.1798 | No |
| 18 | CDK2 | na | 14696 | 0.022 | -0.1770 | No |
| 19 | ATM | na | 14715 | 0.022 | -0.1744 | No |
| 20 | TNFRSF10B | na | 15297 | 0.016 | -0.1889 | No |
| 21 | SHISA5 | na | 16041 | 0.008 | -0.2092 | No |
| 22 | ATR | na | 16093 | 0.008 | -0.2095 | No |
| 23 | SFN | na | 16425 | 0.004 | -0.2185 | No |
| 24 | TP53I3 | na | 16796 | 0.001 | -0.2290 | No |
| 25 | CD82 | na | 17019 | -0.002 | -0.2352 | No |
| 26 | PMAIP1 | na | 17628 | -0.007 | -0.2517 | No |
| 27 | PERP | na | 18314 | -0.014 | -0.2695 | No |
| 28 | CASP8 | na | 18593 | -0.016 | -0.2752 | No |
| 29 | CCND1 | na | 18820 | -0.019 | -0.2792 | No |
| 30 | PTEN | na | 19758 | -0.028 | -0.3023 | No |
| 31 | CDK1 | na | 20745 | -0.037 | -0.3255 | No |
| 32 | CASP9 | na | 20912 | -0.039 | -0.3248 | No |
| 33 | CDKN2A | na | 21184 | -0.042 | -0.3267 | No |
| 34 | PPM1D | na | 21458 | -0.045 | -0.3283 | No |
| 35 | BBC3 | na | 21579 | -0.046 | -0.3253 | No |
| 36 | BAX | na | 22034 | -0.051 | -0.3312 | No |
| 37 | GADD45G | na | 22182 | -0.053 | -0.3280 | No |
| 38 | SESN2 | na | 22271 | -0.054 | -0.3230 | No |
| 39 | CYCS | na | 22416 | -0.055 | -0.3194 | No |
| 40 | DDB2 | na | 22664 | -0.058 | -0.3184 | No |
| 41 | CDK6 | na | 23374 | -0.067 | -0.3295 | No |
| 42 | CCNB3 | na | 23783 | -0.072 | -0.3312 | No |
| 43 | CDK4 | na | 24008 | -0.075 | -0.3270 | No |
| 44 | CDKN1A | na | 24177 | -0.078 | -0.3209 | No |
| 45 | SERPINE1 | na | 24304 | -0.080 | -0.3133 | No |
| 46 | CCNB1 | na | 24923 | -0.089 | -0.3186 | No |
| 47 | GTSE1 | na | 25448 | -0.098 | -0.3200 | No |
| 48 | CCNB2 | na | 26396 | -0.114 | -0.3312 | Yes |
| 49 | STEAP3 | na | 26518 | -0.117 | -0.3183 | Yes |
| 50 | EI24 | na | 26593 | -0.118 | -0.3039 | Yes |
| 51 | CCND3 | na | 26722 | -0.120 | -0.2907 | Yes |
| 52 | IGFBP3 | na | 26956 | -0.125 | -0.2799 | Yes |
| 53 | CHEK2 | na | 27683 | -0.141 | -0.2810 | Yes |
| 54 | FAS | na | 28081 | -0.151 | -0.2713 | Yes |
| 55 | GADD45B | na | 28272 | -0.155 | -0.2549 | Yes |
| 56 | SESN1 | na | 28368 | -0.158 | -0.2355 | Yes |
| 57 | ZMAT3 | na | 29328 | -0.185 | -0.2371 | Yes |
| 58 | RCHY1 | na | 29948 | -0.204 | -0.2262 | Yes |
| 59 | CCND2 | na | 30134 | -0.211 | -0.2019 | Yes |
| 60 | RRM2B | na | 31129 | -0.247 | -0.1958 | Yes |
| 61 | CCNG2 | na | 31639 | -0.271 | -0.1724 | Yes |
| 62 | CHEK1 | na | 32222 | -0.304 | -0.1464 | Yes |
| 63 | TP53 | na | 32227 | -0.304 | -0.1037 | Yes |
| 64 | RPRM | na | 33425 | -0.398 | -0.0823 | Yes |
| 65 | RRM2 | na | 33544 | -0.411 | -0.0279 | Yes |
| 66 | SESN3 | na | 33755 | -0.441 | 0.0281 | Yes |
Table: GSEA details [plain text format]

  

Fig 2: KEGG\_P53\_SIGNALING\_PATHWAY      
 Blue-Pink O' Gram in the Space of the Analyzed GeneSet

  

Fig 3: KEGG\_P53\_SIGNALING\_PATHWAY: Random ES distribution      
 Gene set null distribution of ES for **KEGG\_P53\_SIGNALING\_PATHWAY**
